# Overcoming Limitations of LoRa Physical Layer in Image Transmission

**DOI:** 10.3390/s18103257

**Published:** 2018-09-27

**Authors:** Akram H. Jebril, Aduwati Sali, Alyani Ismail, Mohd Fadlee A. Rasid

**Affiliations:** 1 Wireless & Photonic Networks Research Center of Excellence (WiPNet), Department of Computer and Communication System Engineering, Faculty of Engineering, UPM, Serdang 43400, Selangor, Malaysia; jebrillakram@gmail.com (A.H.J.); alyani@upm.edu.my (A.I.); fadlee@upm.edu.my (M.F.A.R.); 2 Department of Communication Engineering, Technical College of Civil Aviation & Meteorology, Esbea, Tripoli 84116, Libya

**Keywords:** LoRa, wireless sensor network (WSN), low-power wide-area network (LPWAN), image encryption, peak signal to noise ratio (*PSNR*), structural similarity index measure (SSIM)

## Abstract

As a possible implementation of a low-power wide-area network (LPWAN), Long Range (LoRa) technology is considered to be the future wireless communication standard for the Internet of Things (IoT) as it offers competitive features, such as a long communication range, low cost, and reduced power consumption, which make it an optimum alternative to the current wireless sensor networks and conventional cellular technologies. However, the limited bandwidth available for physical layer modulation in LoRa makes it unsuitable for high bit rate data transfer from devices like image sensors. In this paper, we propose a new method for mangrove forest monitoring in Malaysia, wherein we transfer image sensor data over the LoRa physical layer (PHY) in a node-to-node network model. In implementing this method, we produce a novel scheme for overcoming the bandwidth limitation of LoRa. With this scheme the images, which requires high data rate to transfer, collected by the sensor are encrypted as hexadecimal data and then split into packets for transfer via the LoRa physical layer (PHY). To assess the quality of images transferred using this scheme, we measured the packet loss rate, peak signal-to-noise ratio (*PSNR*), and structural similarity (SSIM) index of each image. These measurements verify the proposed scheme for image transmission, and support the industrial and academic trend which promotes LoRa as the future solution for IoT infrastructure.

## 1. Introduction

Wireless remote sensing (WSN) technologies vary in their ability to transfer data at low powers, high speeds, and over long ranges. Today, low-power wide-area networks (LPWANs) are the ideal WSN technology as their extended coverage, low cost, and energy saving features (which are possible without the need for communication infrastructure [1]) make them complementary to short-range wireless technologies, such as Wi-Fi and Bluetooth Low Energy, and credible alternatives to cellular technology, for urban-scale Internet of Things (IoT) applications. This ability for long-range communication to thousands of devices (star topologies) at a low cost and limited power consumption is due to improvements in duty cycling and networking protocols [2]. Long Range (LoRa), narrowband IoT (NB-IoT), IEEE 802.15.4, Sigfox and LTE-M are examples of recent LPWAN technologies used by (IoT) applications to connect wireless sensors to extend the deployment of the IoT and reduce power consumption.

**Figure 1:**
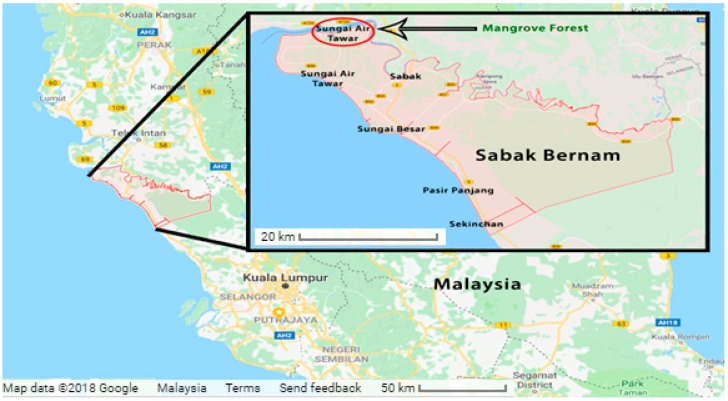
Google map illustrating the location of the target mangrove forest site in Sabak Bernam, Malaysia.

**Figure 2:**
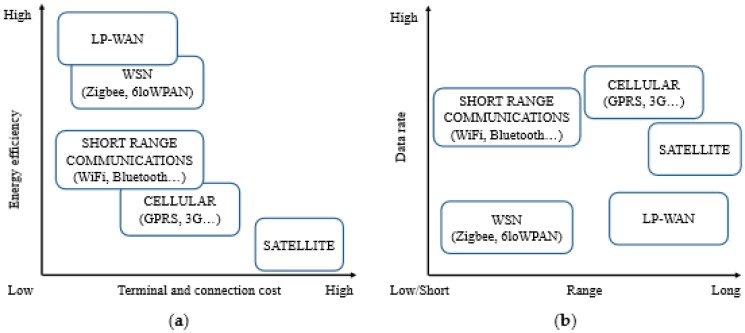
LPWAN is currently the state-of-the-art technology in transmitting images given global concern for low power consumption and limited bandwidth [9]. (**a**) LPWANs are the highest in energy efficiency and lowest in costs. (**b**) LPWANs are the longest in coverage range and the lowest in data rate.

One of the most important features of LPWANs is the ability to enhance the signal-to-noise ratio (SNR) at the receiver by narrowing its bandwidth or by spreading the energy of the signal over a wider frequency range. For example, in NB-IoT, narrowing the bandwidth to less than 25 kHz increases the SNR and enhances the performance of the transceiver. A similar effect is possible with Sigfox when the bandwidth is reduced to approximately 100 Hz. With LoRa, narrowing the bandwidth of a receiver enables the decoding of transmissions with signal amplitudes 19.5 dB below the noise floor, consequently enabling communication at longer ranges than other techniques. The capability for long range communication with LoRa characterizes it as the future network infrastructure for advanced IoT applications, since it can connect hundreds of IoT end-devices to one gateway, and does not need special requirements to connect to other existing networks. In this paper, we demonstrate the first application of LoRa in a WSN for transferring images of mangrove forests captured by an image sensor and used for mangrove monitoring in the Sabak Bernam district of Malaysia (see Figure 1). Currently GPP (2G/3G) technology by Ericsson Co. [3] used to monitor the status of the mangrove trees by reading data from soil moisture, smoke, sea level measurements, and temperature sensors. Subsequently, these data transferred to the community center and processed to develop strategies to prevent the degradation of the mangrove forest. Using LoRa as a WSN with Image sensors for the first time in this area beside it will help in the environment monitoring process, it will contribute in adding LPWAN capabilities of reducing power consumption (powered by batteries for years), cost of installation (no need for more expensive equipment) and increase the range of transferring data (see Figure 2 for more information).

The rest of the paper is structured as follows. First a LPWAN image transferring related works is discussed. Then, a background is provided about LoRa network and some important parameters dictating its operation. After this, a comparison is done between LoRa and other LPWAN technologies which highlights the advantages of this technique for WSN applications. After this, details of the novel image transfer experiment and how it was set up are discussed. We subsequently list and discuss the results of this experiment, to verify the viability of image transfer over the LoRa physical layer. Our results indicate that LoRa is an ideal technology for mangrove monitoring WSN.

## 2. Related Works

Most of the recent research on LoRa network focuses on its outdoor performance and coverage. Significantly, LoRa proved it can deliver more than half of the packets from 5–10 km range in urban applications and in maritime applications where 15–30 km distances observed [4]. A few studies expressed concern regarding image transmission over the LoRa network since images demand a high amount of bits and consume more energy in the transmission operation. One of these significant related works is a paper by Congduc Pham [5] who built an “off-the-shelves low-cost components” image sensor platform which showed promising results of transmitting images over LoRa, although only a 1.8 km range achieved this without packet loss. Another paper by Fan et al. [6] gives a theoretical analysis for the new wireless visual sensor protocol using LoRa modulation but this was not tested outdoors unlike our paper. 

To the best of our knowledge, there is no other study testing LoRa physical layer (PHY) capabilities in images transmission for a mangrove forest area thus far. This is because its long range capability affects the bandwidth which is considered a big barrier in transmitting images and multimedia data [7]. Although, most of the current implemented networks are short range networks, they have a wide bandwidth and high data rate (e.g., Zibgee, LTE, etc.), and they have a serious problem with a limited link budget [8]. Moreover, LoRa and other LPWANs are cheaper in installation and do not require repeaters for distance beside LPWAN are cheaper to install and do not require repeaters for distance. Also, LPWANs require less power consumption (see Figure 2).

In the following section, details about LoRa networks and comparisons with other LPWAN networks are discussed.

## 3. Background

### 3.1. LoRa Network

LoRa, which stands for the initial letters of the words ‘Long Range’, is a combination of communication technologies and protocols, developed by Semtech (Camarillo, CA, USA), that provides significantly longer range than other LPWAN technologies. Modulation with LoRa is based on spread-spectrum techniques, and a variation of a chirp spread spectrum (CSS) modulation integrated with forward error correction (FEC) [10]. LoRa’s CSS is the key factor for long distance signal transfer with a high-quality link, even when the signal power is up to 20 dB lower than the noise floor [11,12]. LoRa uses unique frequency shift keying (FSK) for demodulating signals 19.5 dB below the noise floor. In contrast, other network technologies are typically only capable of demodulating signals with powers of 8–10 dB above the noise floor [10].

The novelty of LoRa is in offering a continuous phase between different chirp symbols in the preamble part of the physical layer packet, which enables simpler and more accurate timing and frequency synchronization, without requiring costly components for generating a settled local clock in the LoRa node [13]. A single LoRa gateway can cover hundreds of square kilometers [14].

The LoRa MAC layer or LoRaWAN is the communication protocol and system architecture layer of a LoRa network. It has the greatest influence on network capacity, quality of service, battery lifetime of individual nodes, and security. To isolate the testing of the LoRa physical layer and its ability to transfer images at a limited bandwidth, LoRaWAN was not used in this paper. 

### 3.2. The LoRa Physical Layer

LoRa can commonly refer to two distinct layers: a physical layer using the CSS and a link layer. The LoRa physical layer consists of many parameters which can be configured into 6720 different settings [15] to offer a wide range of choices for ensuring a good quality link or consuming less energy. The most important parameters of the LoRa physical layer are the carrier frequency (CF), spreading factor (*SF*), bandwidth (*BW*), transmission power (TP), and coding rate (CR). These are discussed further in the proceeding paragraphs, and Table 1 below.

The following are definitions for these parameters:

- Carrier Frequency: CF represents the central transmission frequency used in a band. This can be programmed between 137 MHz to 1020 MHz, in steps of 61 Hz.
- Spreading factor: *SF* is the ratio between the symbol rate and the chip rate. It can be set between 6 and 12 in LoRa [16]. For each *SF*, there are 2*^SF^* chips per symbol. *SF* has a more significant influence on energy consumption than increasing the transmission power, which increases the communication range. Hence, modifying the *SF* is more effective in reducing the energy consumption while maintaining the communication range. A larger *SF* increases the communication sensitivity and data bit rate and reduces the time on air (TOA).
- Bandwidth: *BW* is the range of frequencies available for transmission. A larger *BW* allows transmission at higher data rates but with a shorter TOA and lower sensitivity. In contrast, a lower *BW* enables a higher sensitivity but a lower data bit rate.
- Coding rate: FEC offers protection against interference and can be configured by setting the CR to 4/5, 4/6, 4/7, or 4/8. In LoRa, more robust protection from interference requires a higher CR, which increases the TOA.
- Transmission power: In LoRa, the power required to transmit a specific data packet can be adjusted as appropriate. The relationship between *SF* and the data bit rate in LoRa is defined as follows
  (1) $$R_{b}=SF\times \frac{1}{\left[2^{SF}/BW\right]}\mathrm{bits}/s$$
  where *BW* is the modulation bandwidth in Hz. From Equation (1), we note that spreading factor is directly proportional to the data rate [17].

### 3.3. Comparison of LoRa and Other LPWAN Technologies

In the following sections, we compare LoRa to a number of prominent LPWAN technologies, to demonstrate that it is the best choice for the WSN monitoring mangrove forests in Malaysia.

#### 3.3.1. LoRa versus NB-IoT

NB-IoT is a network technology based on the long-term evolution (LTE) standard that offers connectivity in the 200 kHz spectrum. Most current IoT applications adopt this technology because of its good coverage and low cost of deployment. NB-IoT has been specified under 3GPP release 13 for inclusion within the LTE system.

The advantages of the NB-IoT standard include the ability to deploy the technology using existing LTE networks, a large coverage area, and a high quality of customer service. Similarly, features of the LoRaWAN standard are a long communication range, and thus, coverage area, long time service of user terminals, low cost of network deployment and user terminals, and a high penetrating power. However, the data rates achievable with LoRaWAN are relatively low [18].

Despite the large coverage area and high-quality link offered by NB-IoT even when implemented using existing LTE networks, the cost of the deployment of this technology is expensive compared to LoRa, as a greater cost is incurred in upgrading existing base stations [18].

#### 3.3.2. LoRa versus IEEE 802.15.4

The IEEE 802.15.4 standard offers data rates approaching 250 kbit/s. However, the transmission range greatly depends on the environment. While, for a clear line-of-sight this can reach 1000 m, the range is measured in tens of meters in most cases. Three unlicensed frequency bands are supported by the IEEE 802.15.4 standard: 868 MHz in Europe, 928 MHz in North America, and 2.4 GHz worldwide [19].

In general, LoRa is less sensitive with respect to interference than IEEE 802.15.4g. Research on channelization has proven that it is resilient enough to obtain high packet reception rates (PRR), even with strong interference from IEEE 802.15.4g signals, which are communicated in the same frequency band. The impact of these signals depends significantly on the configuration of the LoRa network (i.e., the *SF* and *BW*). At high data rates, LoRa tolerates interference from signals with amplitudes that are 6 dB higher than the power of the actual LoRa signal being received. At low data rates, this tolerance goes up to 16 dB, as defined by the acceptable PRR [20].

#### 3.3.3. LoRa versus Sigfox

Of the wireless communication technologies that have been classified for IoT applications, the two that are commonly deployed in the ISM band (LoRaWAN and Sigfox) are long range. Sigfox is a proprietary ultra-narrowband technology, developed by an eponymous company based in Labège, France, which uses differential binary phase-shift keying (DBPSK) modulation. In contrast, modulation with LoRa is based on proprietary spread spectrum techniques and Gaussian frequency shift keying [21]. Communication in Sigfox is performed in the 868-MHz frequency band, with the spectrum divided into 400 channels of 100 Hz. Sigfox claims that up to a million end-devices can be handled by only one access point, and the coverage can reach 50 km in rural areas and 10 km in urban areas [12].

With the Sigfox network, data packages are transmitted three times at random frequencies, to maximize the probability of successfully receiving at least one packet, as it is more sensitive to interference than LoRa networks. In [21], coverage and capacity for Sigfox and LoRaWAN on area covering 150 km^2^ of urban areas Northern Denmark tested, and found that uplink indoor coverage (20 dB penetration loss), the impact from interference is even worse, as the link budget is reduced, where LoRaWAN has 78% coverage and Sigfox less than 50% coverage.

#### 3.3.4. Summary of the Advantages of LoRa 

From the comparisons performed in the previous sections, it is evident that LoRa has many advantages over other LPWN networks. The most significant advantages of LoRa with respect to its deployment in future infrastructure networks are [16]

- Orthogonal packet transmission using different SFs (from 6 to 12) concurrently, without data collisions or degradation in performance, reducing TOA.
- Long range communication where outdoor coverage can reach several kilometres, while indoor coverage reaches hundreds of meters and coverage in multi-hop scenario approaches tens of kilometres,
- Low cost of network deployment as nodes can reach the sink directly without intermediary routes,
- Adaptive data rates and multichannel modem transceivers for receiving messages from a large number of network nodes,
- Very low energy consumption for a long battery lifetime,
- High penetrating ability of radio signals; and,
- Operation in the unlicensed range.

### 3.4. Limitations to Image Transmission over LoRa

**Figure 3:**
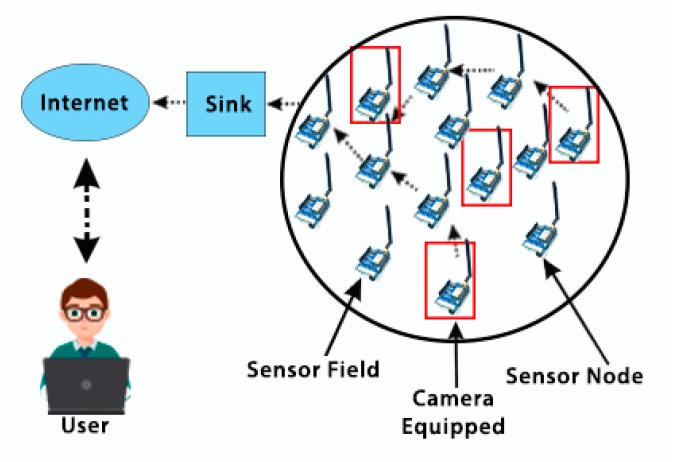
Composition of a wireless sensing network (WSN) [1].

WSNs (see Figure 3) consist of remote sensing nodes monitoring aspects of their environment, which send collected data to a sink center for processing for a specific application. The biggest design concern for WSNs is energy consumption, which should be reduced in order to offer long-range connections and wide coverage. However, the transfer of data from the sensing nodes to the sink is hindered by the narrow bandwidth of these networks and their limited memory for computational processes [1]. Moreover, LoRa technology constrained to 1% duty-cycle (i.e., 36 s/h) applies to the total transmission time, which means only can transmit data 36 s each one hour of transmission time [22]. This limitation makes transferring data from devices like image sensors very complex, as a large bit rate is required for the communication of image data. Hence, although LoRa has the ability to combine the features of LPWA networks and WSN networks, transferring multimedia data over LoRa is a challenging issue because of the data transmission limitation.

The bandwidth of transmission is the most important factor that affects the performance of the CSS technique used for LoRa modulation. A LoRa symbol consists of 2*^SF^* chirps, with one chirp transmitted per second (chirp rate), equal to a bandwidth of one Hertz [12]. Moreover, with LoRa modulation the bit rate and symbol rate at a given *SF* are proportional to bandwidth.

The duration of a LoRa symbol, *T_s_*, can be calculated from the following equation, while the data bit rate can be calculated using Equation (1) [12]:

(2) $$T_{s}=\frac{2^{SF}}{BW}$$

From Equations (1) and (2) we observe that decreasing the bandwidth will decrease the transmission rate and consequently increase the sensitivity. Hence, to improve the performance of LoRa and increase the communication range, the bandwidth must be limited, such that only a smaller bit rate data can be transferred. In this paper, the lowest *BW* configuration (125 kHz) is used to attain the highest sensitivity for long transmissions.

## 4. Experimental Method

**Figure 4:**
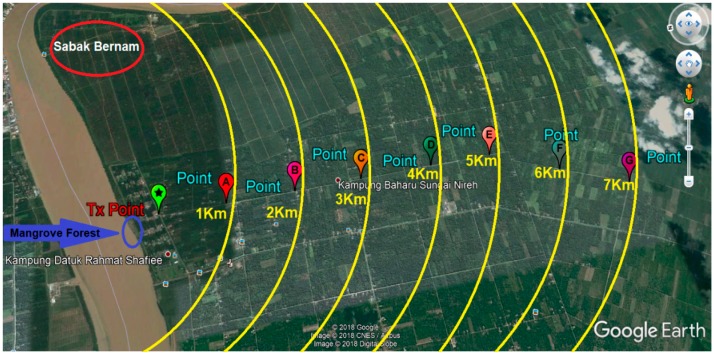
LoRa measurements from different locations in Sabak Bernam (Ptx = 14 dBm).

To verify the feasibility of image transferring over the LoRa physical layer, we conducted a practical measurement experiment in the Sabak Bernam mangrove forest, northwest of the Selangor state, Malaysia. For this experiment, the LoRa transmission node was fixed to the top of a Skylift crane, at a height of approximately 40 m, and the receiver node was installed to the back of a four-wheel drive vehicle, which travelled one kilometer following each measurement, to allow for the assessment of image quality at different distances, as illustrated in Figure 4.

### 4.1. Hardware Setup

**Figure 5:**
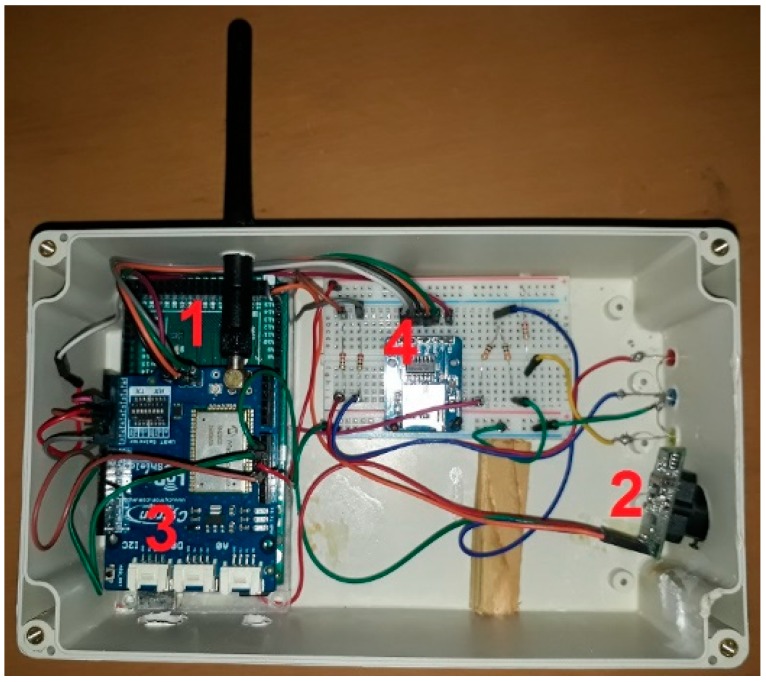
Components of the LoRa transmission node: (**1**) Arduino Mega microprocessor. (**2**) TTL Serial JPEG camera. (**3**) LoRa Shield radio adapter. (**4**) SD Card Module.

**Figure 6:**
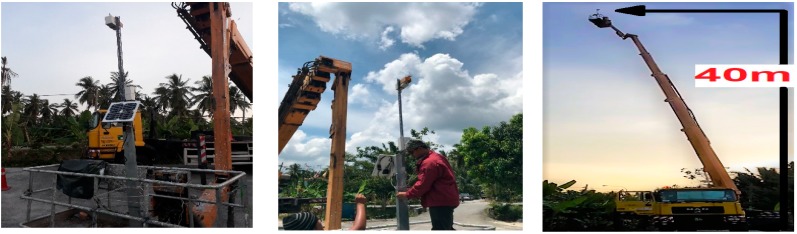
Completed transmission node enclosed in a weatherproof box and fixed on top of a Skylift crane at a height of 40 m.

The transmission node, as illustrated in Figure 5, consists of a LoRa Arduino shield that comprise of an RN2903 transceiver (microchip) stacked on an Arduino MEGA microprocessor. It is good to know that Arduino is an open source board and contains a very simple microcontroller which can be connected to various types of sensors and devices. Furthermore, Arduino consumes less power than other tiny controlling devices such as Raspberry Pi, which is more complicated and considered as fully functional computer [23]. An Adafruit TTL Serial JPEG camera connected to the Arduino board was used for capturing images. Also, a MicroSD card adaptor is connected to save the captured photos. The transmission node circuit is powered by a 12 V battery, which is charged by a solar panel, ensuring the node could be powered at a reduced cost. All these components are enclosed in a weatherproof box, to protect them from adverse environmental conditions, as shown in Figure 6.

**Figure 7:**
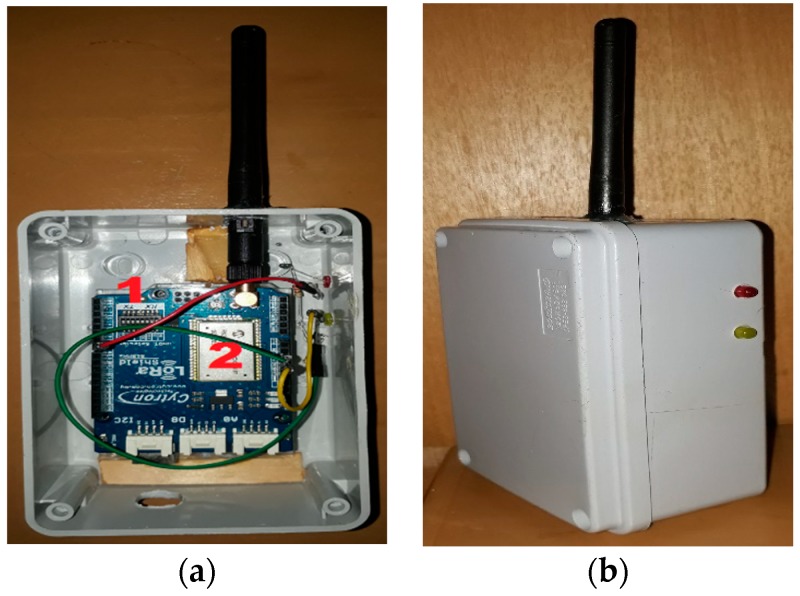
(**a**) Receiver node: (1) Arduino Uno (2) LoRa RN2903 Module. (**b**) The receiver node enclosed inside a weatherproof box.

Similarly, the receiver node (see Figure 7a) consists of a LoRa RN2903 Module controlled by an Arduino Uno microprocessor and stacked on top of it. These components are also enclosed inside a weatherproof box, as shown in Figure 7b.

**Figure 8:**
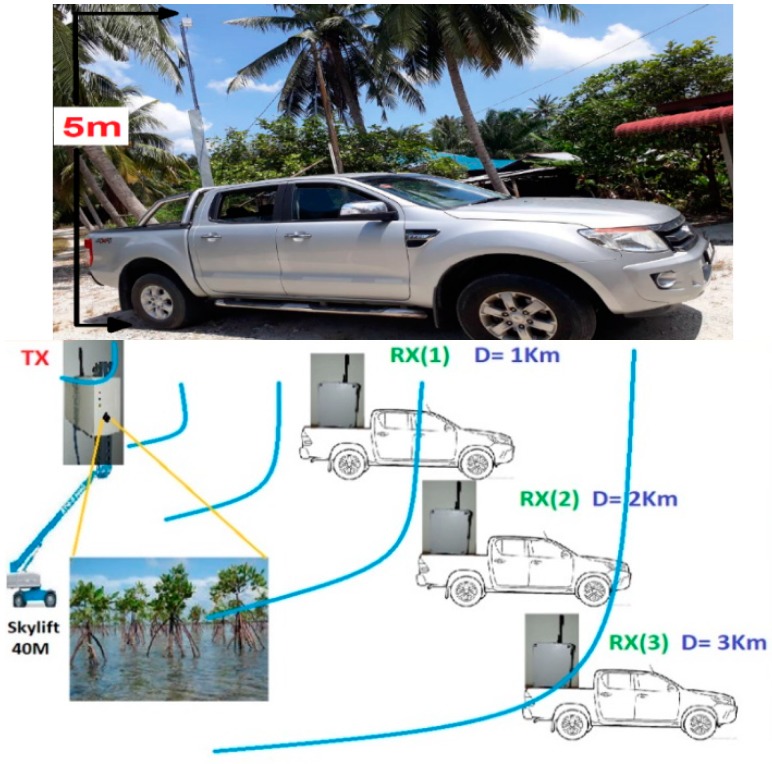
Completed receiver node fixed to the back of a four-wheel drive vehicle to enable testing at different distances.

Figure 8 shows the receiver node installed on the back of the four-wheel drive vehicle in order to test it at different distances.

### 4.2. Experimental Setup

**Figure 9:**
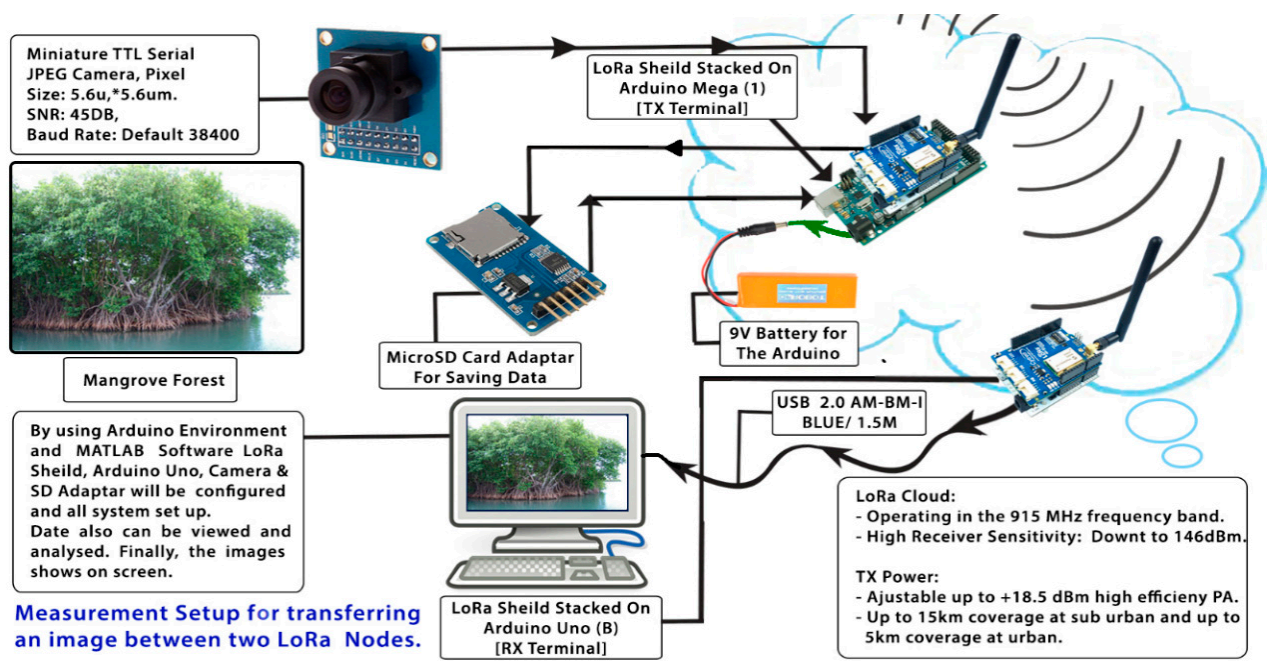
Experimental setup for transferring images of the mangrove forest between two LoRa nodes.

Figure 9 illustrates the operation of the complete test setup. Here, the TTL camera captures a JPEG image of the mangrove trees. After this, the Arduino MEGA microprocessor saves the photo data in the SD memory and coverts this data into a hexadecimal format. The hexadecimal data is then transferred to the receiver node via the LoRa physical layer. This data is split into 84-character packets to enable transmission. Once all these packets have been received, they are sent by the Arduino Uno processor to a personal computer, where they are reassembled and displayed using MATLAB code.

#### 4.2.1. Image Encryption Scheme for LoRa Transmission

Data transmission from high bit rate devices, such as image sensors, over the LoRa network is very slow. Such information is inappropriate for transfer with this technology because of the bandwidth limitation and LoRa constrained to 1% duty-cycle (i.e., 36 s/h) which means only can transmit data 36 s each one hour of transmission time. The LoRa MAC layer is typically responsible for transferring data from nodes to gateways. However, it has a limited ability to cope with image data due to the typical image size and the encryption used in the MAC layer. Hence, to enable transmission of image data, the LoRa physical layer is used with a new encryption method instead (see Appendix A). 

With the new method the image is captured by the Adafruit TTL camera and then saved in SD memory in the JPEG file format. From there it is subsequently converted into a hexadecimal formatted file. The Arduino processor then splits the hexadecimal file into packets containing 84 hexadecimal characters, which is the largest file size that can be transferred over the LoRa bandwidth at one time. To begin serial data transfer, the LoRa radio transmission command ‘radio tx’ is sent to the receiver node, followed by the hexadecimal packet. Data transfer is paused until the LoRa receiver node sends an acknowledgement that the initial packet has been received successfully. Then, subsequent packets are transmitted.

#### 4.2.2. Receiving Images over LoRa

The LoRa radio adapter in the receiver node listens for transmitted signals (see Appendix A) and sends a receipt of acknowledgement when they successfully arrive. Once all hexadecimal packages have been received, they are collected into one data variable by the Arduino processor and then sent, through a serial connection, for processing in MATLAB in order to retrieve image data from the hexadecimal package.

#### 4.2.3. MATLAB Manipulation of Received Images

**Figure A5:**
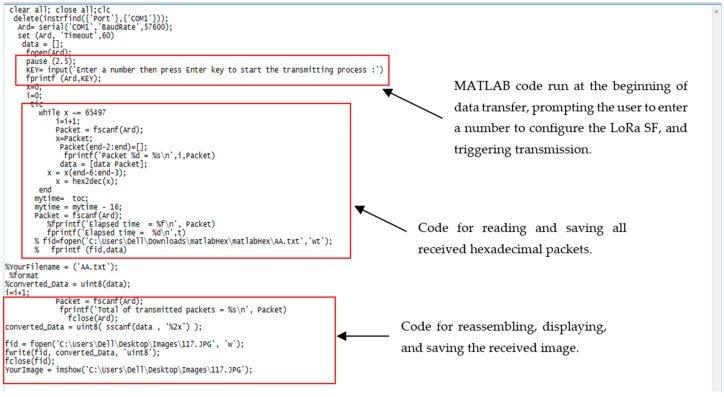
The MATLAB controls for initiating the image capture process and reassembling image files from transmitted hexadecimal packets.

MATLAB is used to help control the process of sending and receiving data through the LoRa adapters and to analyse the quality of the images before and after transfer over the LoRa network, using the *PSNR* and *MSE*. Prior to transmission, the MATLAB code sends a prompt message asking the user to enter a number from 1 to 6 to trigger transmission. These numbers correspond to the settings for the *SF* which, in this case, vary from 7 to 12. Following this, the data transfer process described above is initiated. The MATLAB code for triggering data transfer, retrieving received data packets, and saving and displaying the reassembled image is highlighted in the Appendix A (Figure A5).

### 4.3. LoRa Physical Layer Settings

The configuration of the LoRa radio parameters for the transmitter and receiver nodes are summarized in Table 2, where a bandwidth of 125 kHz is used as the narrowest *BW* value that can be configured by the LoRa RN2903 Module. The aim of using this value is to ensure the highest sensitivity rate and long communication range. The used values of *SF* ranges from 7 to 12. Those values can be chosen by the default settings recommended by Semtech, the LoRa developer.

### 4.4. Metrics

The peak signal-to-noise ratio (*PSNR*) is typically used to assess the quality of an image transmitted over a network. As image quality is assessed quantitatively, this based on the difference between the pixels of the image reconstructed following transmission and the original image [24].

The *PSNR* of a transmitted image can be calculated as

(3) $$PSNR=10\log\frac{S^{2}}{MSE}$$

where, for an 8-bit image, *S* is 255 and *MSE* is the mean squared error, which is the average of the squared difference in the intensity pixels in the original image and the output image [24]. This is calculated as

(4) $$MSE=\frac{1}{mn}\overset{m-1}{\underset{i=0}{\sum }}\overset{n-1}{\underset{j=1}{\sum }}{\left[I\left(i,j\right)-K\left(i,j\right)\right]}^{2}$$

where *m* and *n* are the respective length and width of the image in pixels *I*(*i*,*j*) and *K*(*i*,*j*) are functions describing the intensity of individual pixels in the transmitted and received image, respectively.

Studies comparing reconstructed images with original images using PSNR and MSE have reported that these two metrics are not sufficient for image quality assessment. It is advised that another metric called the ‘structural similarity index’ (SSIM) is used in addition to these, to measure the degree of similarity between the original and the transmitted image. Hence, in testing the ability of LoRa to transmit images using the proposed method, we used these three metrics to define the transmission quality.

## 5. Results

In this section, we consider the results of the data transfer of captured mangrove forest images over the LoRa physical layer using different SFs and at different distances. In addition to the quality assessment metrics, we also consider packet loss and the effect of the surrounding environment on transmission.

### 5.1. Packet Loss in LoRa Transmission

Table 3 shows a series of characteristics that define the success of image transmission over the LoRa physical layer. With this set of experiments, the *SF* was varied from 7 to 12, and communication ranges between 1 and 7 km were tested. In this table, we record the number of hexadecimal packets received, the packet loss ratio, and the time elapsed for data transfer (calculated using additional code in the receiver node) for each *SF* and distance setting.

**Figure 10:**
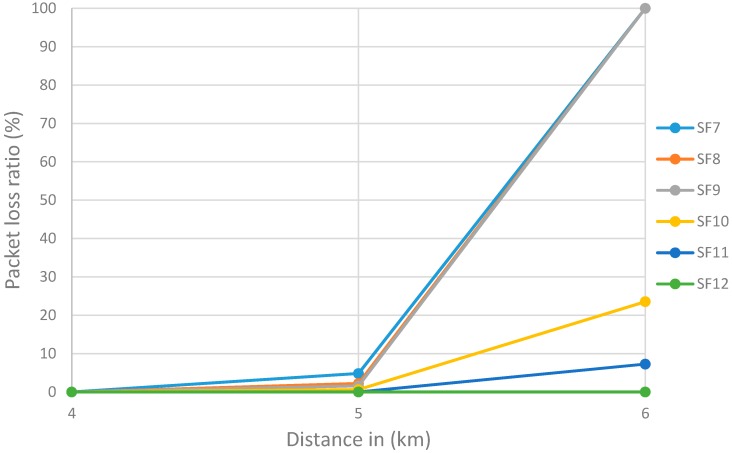
Correlation between *SF* and packet loss ratio.

Remarkably, we observed no packet loss between 1 and 4 km, when *SF* = 7 (see Table 3 above) where time elapsed during data transfer was less than 2 min. We also observed that 4.82% of the packets were lost when the image was transferred over a 5 km range. All packets were lost when transferring at ranges of 6 km and above. Similarly, no packet loss was observed at distances shorter than 4 km when *SF* = 8, 9 and 10. The communication range without packet loss increased to 5 km when *SF* = 11 and 6 km when *SF* = 12. With the latter *SF*, a packet loss of 2.82% was observed (see Table 3 above). Most important notice that an *SF* of 7 reduces the time elapsed during transfer, and *SF* 12 is preferable for long distance transmission. At the end 12 images out of 21 were received successfully (see Table 4) and most remarkable results that SF7, SF8, and SF9 were very close, although the measurements were in different distances and this is clearly shown in Figure 10.

Visual inspection of the images before and after transfer over the LoRa network (see Table 5) indicate the success of the new image encryption scheme, although some of the received images were affected by noise. By splitting this ‘high bit rate data’ into packets, we are able to overcome the LoRa bandwidth limitation. 

### 5.2. Quality Assessment of Received Images

After reassembling the received images into the JPEG file format, we calculated the values of *PSNR*, *MSE*, and SSIM for the different transmission conditions using MATLAB, for image quality assessment. The results of these calculations are summarised in the following table.

We observed from Table 6, that the values of *MSE* and SSIM were 0 and 1, respectively, at communication distances between 0 and 3 km when *SF* was set to 7. These values indicate that the original and the received images were identical in these transmission conditions. The same values were calculated for images transmitted with SFs of 10, 11, and 12 at a distance of 4 km from the transmission node. The following table (Table 7) summarizes the *SF* settings where the original and transmitted image are identical.

### 5.3. Effects of Fresnel Zone

**Figure 11:**
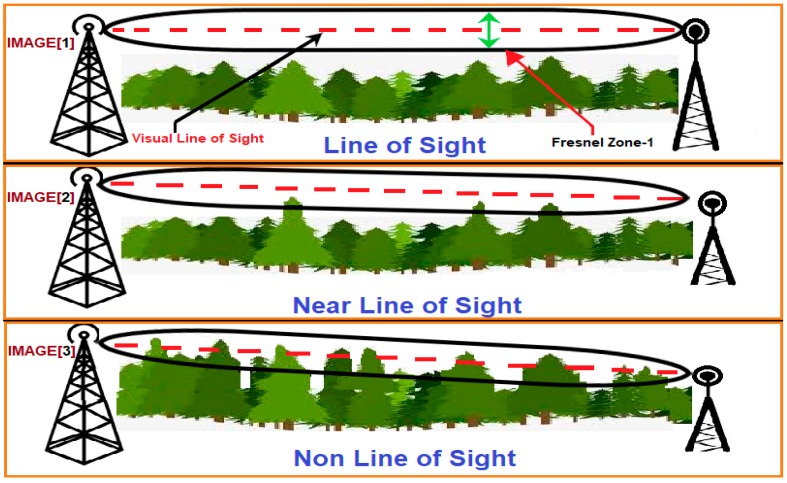
Illustration of line-of-sight point-to-point wireless communication. Image 3 shows how the trees act as obstacles that can affect signal performance.

**Figure 12:**
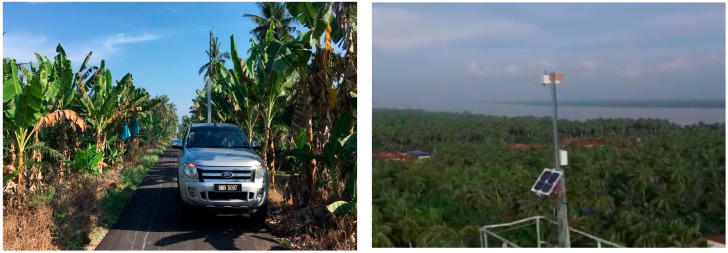
Dense growth of Coconut trees around the mangrove forest, which fall in the first order Fresnel zone.

Although the results of this experiment demonstrate that by adopting the proposed encryption scheme image data can be transferred over the LoRa physical layer, they do not indicate the full capabilities of LoRa communication based on the nine trials which ended in failure. We posit that the reason for these failures was the dense growth of coconut trees around the mangrove area, which worked as obstacles preventing signals from traveling between the transmitter and receiver nodes. These trees lie in the so-called ‘Fresnel zone’, defined in point-to-point networks by a cylindrical ellipse drawn between the transmitter and the receiver nodes, as illustrated in Figure 11. It is known that this zone should be at least 60% free of obstructions, such as buildings, mountains, or trees, failing which, signal energy loss is incurred. This, reduce the performance of the wireless link, as was the case with our experiment. Hence, it is important to have a clear ‘line of sight (LOS)’ between the transmitter and the receiver, as much as possible [25]. In our experiments, the transmitter node was fixed on a Skylift at a height of 40 m. The height of the receiver node, which was installed on the back of a four-wheel drive vehicle did not exceed 5 m. On average, coconut trees are approximately 12 m tall. This height can sometimes approach 25 m in isolated incidences [26]. Consequently, these trees fall in the first order Fresnel zone, based on the geometry of the measurement environment, as illustrated in Figure 12. This has a negative effect on the transmission of the LoRa signals.

To figure out whether or not the trees are acting as an obstacle of signal performance between the sender and the receiver, the Fresnel zone radius and diameter can be calculated using the following formulas:

(5) $$r=8.656\times \sqrt{\frac{D}{F}}$$

where

*r* = The radius of the Fresnel zone
*D* = The distance between Rx and Tx (km)
*F* = Frequency used in the transmission process which is 921.9 MHz

Fzone Diameter = *r* × 2 (6)

The Fresnel zone midpoint calculated using the following

(7) $$\mathrm{Fzone}\;\mathrm{midpoint}\;\mathrm{height}=\frac{40+5}{2}=22.5\;m$$

where the 40 m and the 5 m are the height of the transmission and the receiver node from the ground respectively. 

The average height of the mangrove trees (coconut trees) were calculated because it useful to find out how much it can encroach through the Fresnel zone, so in our experiment they calculated this using the following equation

(8) $$\mathrm{Averge}\;\mathrm{Height}\;\mathrm{of}\;\mathrm{Coconut}\;\mathrm{trees}=\frac{25+12}{2}=18.5\;m$$

where the 12–25 is the range of coconut trees heights

For example, the Fresnel zone for the first kilometre distance between the transmission and receiver nodes can be calculated using the above Equations (5)–(8) as the following

$$r=8.656\times \sqrt{\frac{1\times 10^{3}}{921.9\times 10^{6}}}=9.01\;m$$

Fzone Diameter = 9.01 × 2 = 18.02 m

Fresnel zone centre height = Fzone midpoint height − Fzone Radius = 22.5 − 9.01 = 13.49 m

Subtracting this value from the average height of coconut trees roughly gives us the distance of the coconut trees which crossed the Fresnel zone.

18.5 − 13.49 = 5.01 m

After this, it is easy to find out how much the Fresnel zone clearance is using the following calculations

$$\mathrm{Percentage}\;\mathrm{of}\;\mathrm{the}\;\mathrm{zone}\;\mathrm{that}\;\mathrm{is}\;\mathrm{non-free}=\frac{5.01}{18.02}\times 100=27.8\%$$

Percentage of the Fresnel zone that is free = 100 − 27.8% = 72.2%

**Figure 13:**
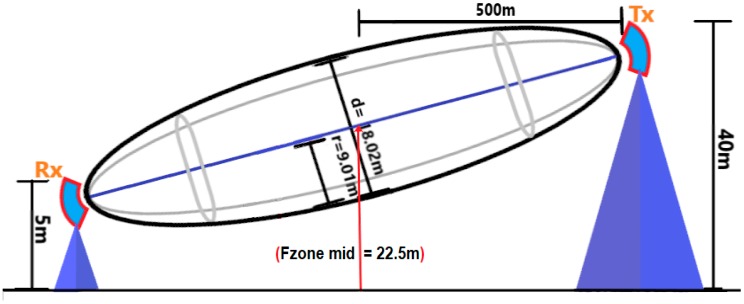
The calculation of the Fresnel zone between the transmitter and the receiver at the first distance (1 km).

Thus, at the first distance (1 km) between the transmitter and the receiver, the Fresnel zone was more than 60% clear of obstruction. This means the coconut trees will not affect the clear line of sight between the transmitter and the receiver (see Figure 13). 

After applying the same calculations for the rest of the distances, the following results were found (see Table 8):

It is obvious from these results (Table 8) that the clear Fresnel zone is less than 60% from the 5th km and above, where the wireless link performance will definitely be affected [27]. Therefore, the coconut trees at the place of measurement and were an obstruction in this scenario and prevent the clear line-of-site status between the transmitter and the receiver nodes. This is the reason for packet loss in the experiment. 

## 6. Conclusions

Using LoRa as the main infrastructure for mangrove monitoring offers longer range connections, reduced costs (as the technology is free to use and the longer range of communication reduces the number of gateways required), and low power consumption, in line with the environmental goals of the mangrove monitoring project. In this paper, we demonstrated the concept of point-to-point data transfer by transmitting images over the LoRa physical layer. This transfer was achieved despite the limited bandwidth of LoRa, using a novel image encryption technique. Our demonstration highlights LoRa as an ideal technology for implementing a WSN for monitoring mangrove forests, which will aid authorities, researchers, and farmers in their efforts to restore dwindling mangrove plantations.

## Figures and Tables

**Table 1:** LoRa parameter settings and their effects on communication performance [2].

Setting	Values	Effects
Bandwidth	125, …, 500 kHz	A higher bandwidth is required for transmitting data at high rates (1 kHz = 1 kcps). However, increasing this parameter decreases the communication range and sensitivity.
Spreading Factor	2^6^, …, 2^12^ $\frac{\mathrm{chips}}{\mathrm{symbol}}$	A higher spreading factor (*SF*) increases the communication range, radio sensitivity, and the signal-to-noise ratio (SNR). However, energy consumption consequently increases.
Coding Rate	4/5, …, 4/8	Bigger coding rates increase the protection against decoding errors and interference bursts at the expense of longer packets and higher power consumption.
Transmission Power	−4, …, 20 dBm	The signal-to-noise ratio is increased by increasing the transmission power at the cost of energy expenditure.

**Table 2:** Settings of the LoRa physical layer during testing.

*BW*	*SF*	PT	CR
125 kHz	From 7 to 12	14	4/8

**Table 3:** Packet loss measurements.

*SF* Setting	Distance (km)	Received Image	Number of Transmitted Packets	Number of Received Packets	Packet Loss Ratio	Elapsed Time (Average)
7	1	Y	314	314	0 P (0%)	1 min + 7 s
7	2	Y	315	315	0 P (0%)	1 min + 7 s
7	3	Y	317	317	0 P (0%)	1 min + 7 s
7	4	Y	312	312	0 P (0%)	1 min + 7 s
7	5	N	311	296	15 P (4.82%)	1 min + 7 s
7	6	N	309	0	309 P (100%)	1 min + 49 s
8	4	Y	312	312	0 P (0%)	1 min + 49 s
8	5	N	316	309	7 P (2.21%)	1 min + 49 s
8	6	N	310	0	310 P (100%)	1 min + 49 s
9	4	Y	318	318	0 P (0%)	3 min + 13 s
9	5	N	315	310	5 P (1.58%)	3 min + 13 s
9	6	N	310	0	310 P (100%)	3 min + 13 s
10	4	Y	309	309	0 P (0%)	5 min + 48 s
10	5	N	311	309	2 P (0.64%)	5 min + 48 s
10	6	N	310	237	73 P (23.54%)	5 min + 48 s
11	4	Y	313	313	0 P (0%)	8 min + 50 s
11	5	Y	313	313	0 P (0%)	8 min + 50 s
11	6	N	316	293	23 P (7.27%)	8 min + 50 s
12	4	Y	317	317	0 P (0%)	14 min + 27 s
12	5	Y	315	315	0 P (0%)	14 min + 27 s
12	6	Y	318	318	0 P (0%)	14 min + 27 s
12	7	N	319	310	9 P (2.82%)	14 min + 27 s

**Table 4:** Summary of the numbers of successfully and unsuccessfully received images.

Total	Y	N
21	12	9

**Table 5:** Visual comparison between original and transferred images for each *SF* setting used in testing.

Original	Received	Original	Received
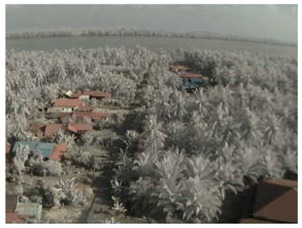	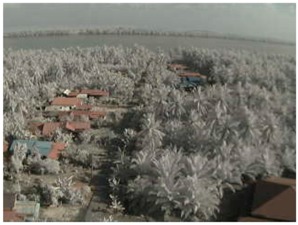	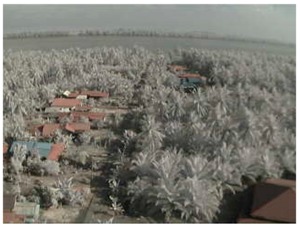	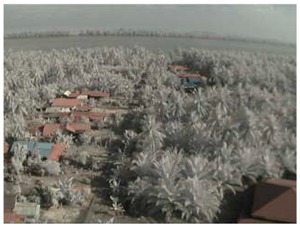
D = 1 km, *SF* = 7		D = 2 km, *SF* = 7	
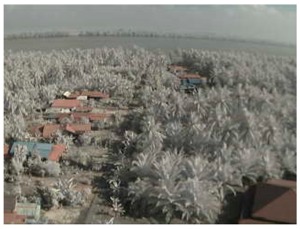	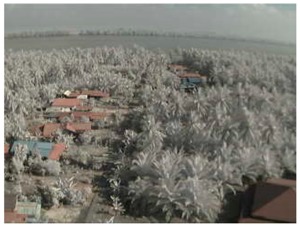	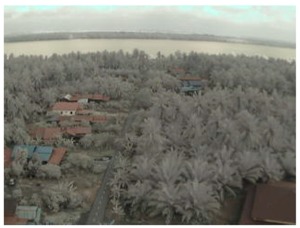	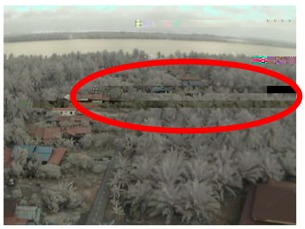
D = 3 km, *SF* = 7		D = 4 km, *SF* = 7	
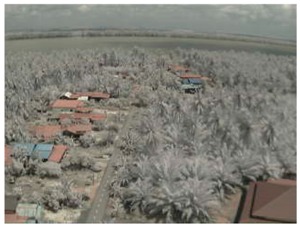	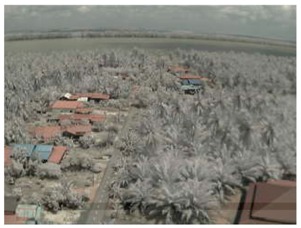	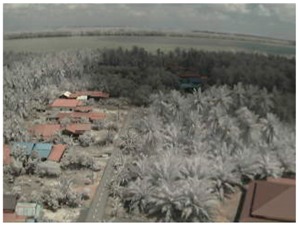	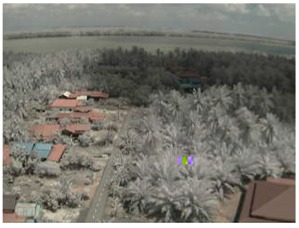
D = 4 km, *SF* = 8		D = 4 km, *SF* = 9	
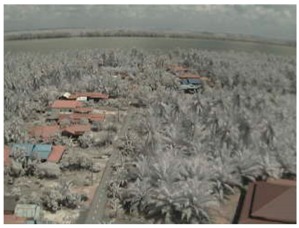	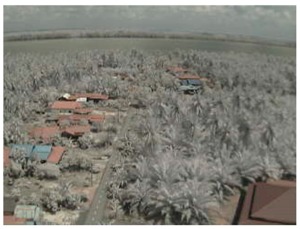	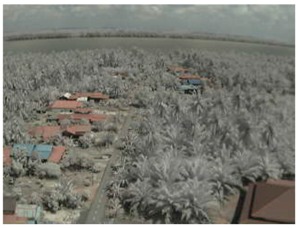	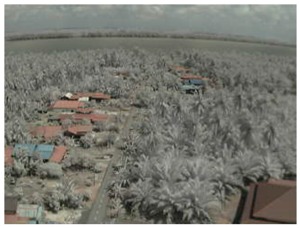
D = 4 km, *SF* = 10		D = 4 km, *SF* = 11	
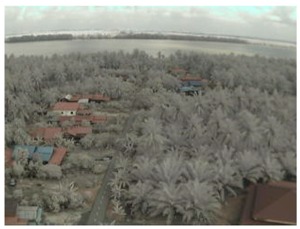	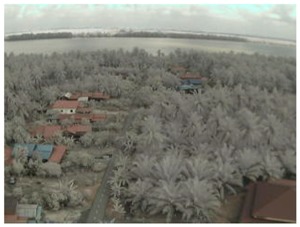	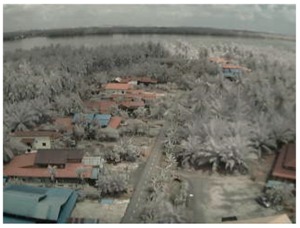	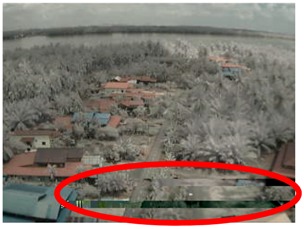
D = 4 km, *SF* = 12		D = 5 km, *SF* = 11	
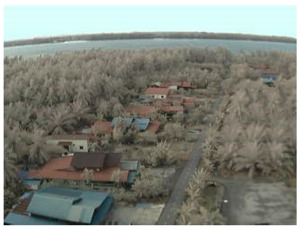	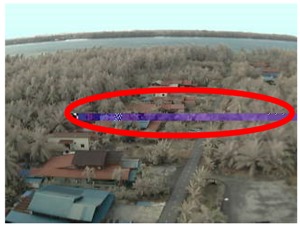	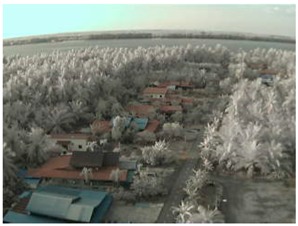	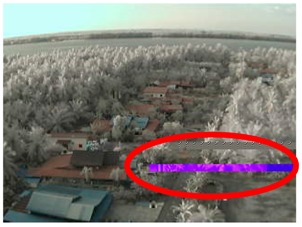
D = 5 km, *SF* = 12		D = 6 km, *SF* = 12	

**Table 6:** Summary of image quality metrics (*MSE*, *PSNR*, and SSIM) calculated using MATLAB.

*SF*	Distance (km)	*MSE*	*PSNR* (dB)	SSIM
7	1	0	INF	1
7	2	0	INF	1
7	3	0	INF	1
7	4	98.7301	28.1863	0.9321
8	4	9.4572	38.3732	0.9953
9	4	8.9987	38.5890	0.9968
10	4	0	INF	1
11	4	0	INF	1
11	5	189.3394	25.3584	0.9437
12	4	0	INF	1
12	5	62.4792	30.1734	0.9625
12	6	234.5309	24.4288	0.9528

INF refers to infinity. We obtained this when the value of a divisor was small, such that the result of the subsequent MATLAB calculation was numerically undefined.

**Table 7:** LoRa physical *SF* setting which has identical image quality metrics.

*SF*	Distance (km)	*MSE*	*PSNR* (dB)	SSIM
7	1 to 3	0	INF	1
10	4	0	INF	1
11	4	0	INF	1
12	4	0	INF	1

**Table 8:** Fresnel zone calculations for the measured distances.

Distance (km)	Fresnel Zone Radius (m)	Fresnel Zone Diameter (*r* × 2)	Percentage of Clear Fresnel Zone
1	9.01	18.02	72.2%
2	12.75	25.50	65.69%
3	15.61	31.22	62.82%
4	18.03	36.06	61.4%
5	20.16	40.32	59.3%
6	22.08	44.16	59.06%

**Figure A1:**
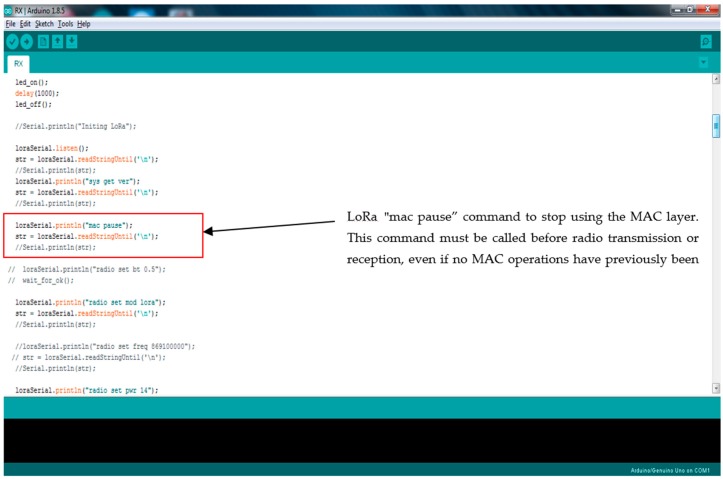
The “MAC pause” command used to stop data encryption over the MAC Layer.

**Figure A2:**
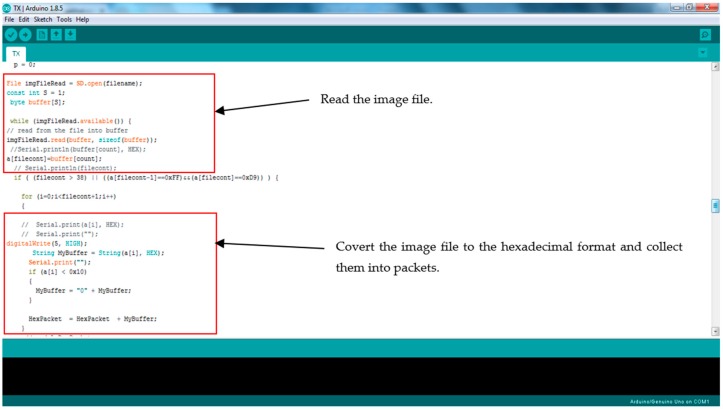
Arduino code for converting the captured image into hexadecimal packets.

**Figure A3:**
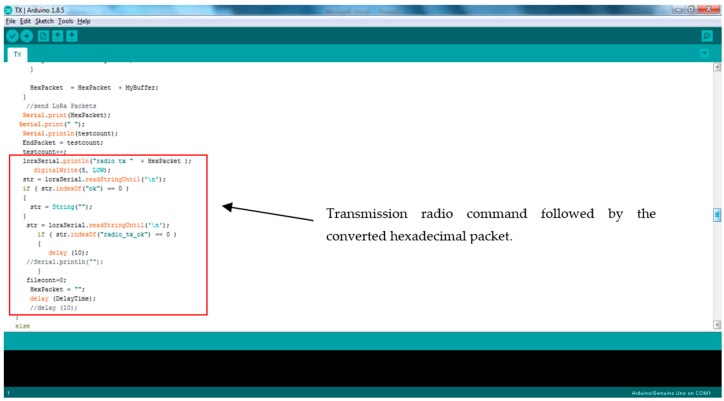
LoRa command for transmission of the hexadecimal packets.

**Figure A4:**
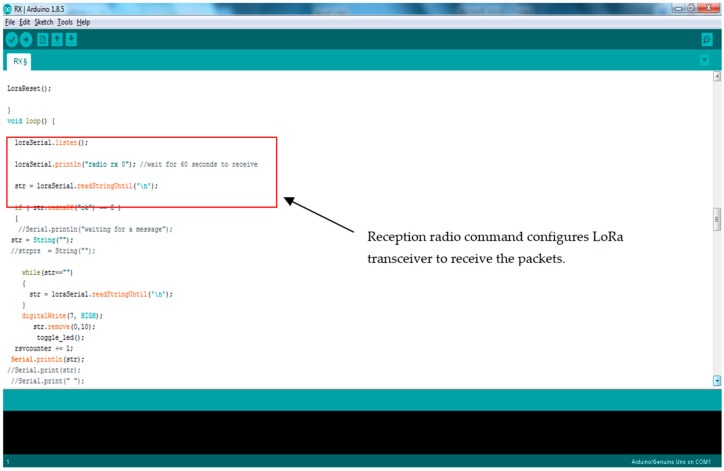
LoRa command preparing the receiver node to listen for incoming data.
